# Supplementary material for: The rising complexity and burden of multimorbidity in a middle-income country
Source: PLoS One. 2020 Dec 11;15(12):e0243614. doi: 10.1371/journal.pone.0243614 (PMC7732070; doi:10.1371/journal.pone.0243614)
Supplement: S1 File — (DOCX) [file pone.0243614.s003.docx]

**Logistic Regression Analysis - INTERPRETATION**

The main objective of using regression modelling in this study was to identify how the variables age, gender, care setting and the presence of multimorbidity of the patients affected the development of mental disorders such as depression, anxiety and/or dementia. The outcome of interest being a binary variable (Yes/No) stating if the patient suffers from a mental disorder(s) or not, it was decided to use a binary logistic regression model.

It is important to carry out the goodness of fit tests necessary to check the major assumptions in logistic regression which will be essential to build a good model while checking how well the model agrees with the observed data.

After fitting the model to the data using the “glm” function in R software (x64 3.0.3) and RStudio (Version 1.3.959), the adjusted odds ratios with their 95% confidence intervals were obtained as shown in the table below. Here, the adjusted odds ratios have been used since they control for confounding bias which means it measures the association between the outcome variable and a confounding variable and controls for that value.

| **Any mental health disorder** | | | |
| --- | --- | --- | --- |
|  | **Standardized Coefficients**  **(95% CI)** | **Adjusted OR**  **(95% CI)** | **p-value** |
| Age (in years) | 0.21  (0.19 , 0.24) | 1.00  (0.98 , 1.03) | 0.801523 |
| Male (Vs Female) | 0.34  (-0.25 , 0.93) | 1.13  (0.63 , 2.03) | 0.686747 |
| Tertiary Care (Vs Primary Health Care) | 0.31  (-0.28 , 0.91) | 1.12  (0.62 , 2.02) | 0.713546 |
| **Multimorbidity**  **(number of disorders)** | **1.98**  **(0.9 , 2.85)** | **7.25**  **(5.82 , 8.68)** | **0.000242** |

The odds ratio values describe the model as it is applied to the data but if the model and the data are not in good agreement, then these odds ratios are not very meaningful. Hence, it is crucial to assess the validity of logistic regression results.

**Logistic Regression Assumptions & Goodness of Fit Diagnostics**

- *Assumption of appropriate outcome structure* - Binary logistic regression requires the dependent variable to be binary which is one of the main assumptions. In this case the dependent variable being dichotomous (YES/NO) satisfies this assumption.

- *Assumption of observation independence* - The observations are required to be independent of each other which means that they should not come from repeated/paired data. This requirement is met in our study with the data consisting of individual patient records.
- *Linearity assumption* - To check the linear relationship between continuous predictor variables and the logit (log odds) of the outcome. The Box-Tidwell Test was used to check this assumption by testing whether the logit transform is a linear function of the predictor, effectively by adding the non-linear transform of the original predictor as an interaction term to test if this addition made no better prediction. Basically in this scenario, the only continuous predictor being ‘Age’, the interaction Age * ln (Age) was added to the regression model and checked if the coefficient became significant. The results showed that the interaction term was non-significant (p-value = 0.240 > 0.05) which thus proved that the linearity assumption is satisfied.

The Hosmer and Lemeshow Test evaluates whether the logistic regression model is well calibrated. For the model to be concluded as well calibrated, the test should result with a non-significant Chi-square result. Thus, at a confidence level of 95%, the following output of the test represents that the adjusted model is well calibrated (p-value = 0.401 > 0.05) which shows that the model satisfactorily fits the whole set of observations.

| **Hosmer and Lemeshow Test** | | | |
| --- | --- | --- | --- |
| Step | Chi-square | df | Sig. |
| 1 | 8.344 | 8 | .401 |

- *Assumption of the absence of Influential* Values - To check if there are influential/extreme values or outliers in the continuous predictors. These problematic values with an unexpectedly large impact on model results could cause the observed value and the model prediction to be in poor agreement. To check this assumption, we have used Cook’s Distance where values exceeding one will show that they may be unduly influencing the model. The Cook’s Distance values were obtained using SPSS (Version 20) software and were arranged in the ascending order to check if there are any values over one. So the highest was resulted as 0.58 which is below 1 showing that there aren’t any unnecessarily problematic points in the data set to be concerned about.
- *Assumption of the absence of Multicollinearity* - There is little/no high multicollinearity (inter-relations) among the predictor variables which means that the independent variables should not be highly correlated with each other. To test this, VIF (Variance Inflation Factor) values are used and a value of VIF > 10 will indicate multicollinearity is present and the assumption is violated. As resulted in the output below having all the VIF values are below 10, it can be concluded that the assumption of the absence of multicollinearity is satisfied.

| **Variance Inflation Factors** | |
| --- | --- |
| **Variable** | **VIF** |
| Age | 1.011381 |
| Gender | 1.077011 |
| Setting | 1.085509 |
| Multimorbidity | 1.000361 |
