# Supplementary material for: The rising complexity and burden of multimorbidity in a middle-income country
Source: PLoS One. 2020 Dec 11;15(12):e0243614. doi: 10.1371/journal.pone.0243614 (PMC7732070; doi:10.1371/journal.pone.0243614)
Supplement: S2 File — (DOCX) [file pone.0243614.s004.docx]

**Multiple Linear Regression - Multimorbidity Study**

Model Building using the variables Age, Gender, Mental Disorders and Number of Disorders.


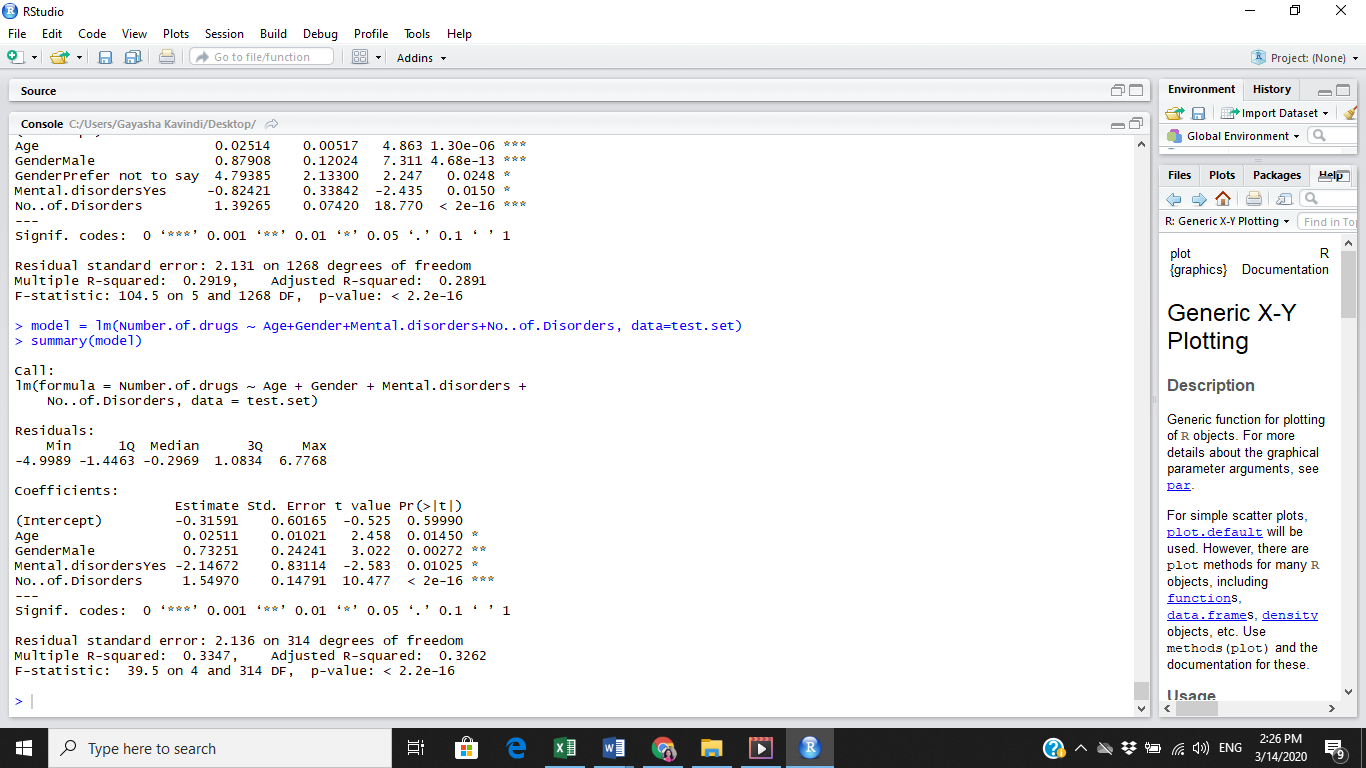


- Overall P - Value on the basis of F-statistic < 0.05

This indicates that the overall model is significant at 95% significance level.

**Testing the model diagnostics underlying regression modelling**

1. **Assumption 1 - Linearity of Relationship**

The residuals appear to form an equal spread around the horizontal line without distinct patterns. Therefore the first assumption is satisfied.

### Assumption 2 - Independence of Variables

The explanatory variables used were found to be independent of one another using correlations and associations.

1. **Assumption 3 - Normal Distribution of Residuals**

The Normal Q-Q plot is used to visually determine if the standard residuals are normally distributed.

1. **Assumption 4 - Homoscedasticity or Equal Variance of Variables**

Explanation:

In R,  ‘e’ stands for 10.

So it is a matter of powers.

So 2e-16 = 2*10^(-16)

The number of drugs was taken as a numerical variable although it was categorized for analysis purposes.

▪One reason for this was since taking the original variable for modeling is better in case if any variation was left out after categorizing.

▪The other was if we take the response variable as a categorical one we would have got to do a multiple logistic regression which would be a bit more complicated with the higher number of categories.
