# Supplementary material for: The rising complexity and burden of multimorbidity in a middle-income country
Source: PLoS One. 2020 Dec 11;15(12):e0243614. doi: 10.1371/journal.pone.0243614 (PMC7732070; doi:10.1371/journal.pone.0243614)
Supplement: Attachment — Submitted filename: Response to reviewers 05.09.2020.docx [file pone.0243614.s005.docx]

**Response to reviewers – Rebuttal letter and table of rectifications**

06/09/2020

Dr Chamath Fernando

Family Physician/Lecturer, Department of Family Medicine

Palliative Care Lead/ Coordinator, National Centre for Primary Care and Allergy Research

Faculty of Medical Sciences

University of Sri Jayewardenepura

Sri Lanka

WHO Primary Health Care Young Leader

Editor-in-chief,

PLOS ONE Journal.

Dear Sir/Madam,

**Submission of the revised article entitled “The rising complexity of Multimorbidity in a middle income country” in view of publication - *Research Article***

We gratefully appreciate the valid comments provided by you and the reviewers on the manuscript that undoubtedly adds much value to the article. We have made the necessary corrections, clarifications and rectifications which are listed in the following table. Along with the rebuttal letter, we are submitting the manuscript with tracked changes as well as an unmarked version of the same.

Kindly advise on how we could improve the article further and do not hesitate to contact me for any further clarifications.

Thank you,

Yours faithfully,

**Dr G.V.M. Chamath Fernando –** *Corresponding author*

+94 76 660 9192

[chemetf@sjp.ac.lk](mailto:shamini@sjp.ac.lk)

|  | **Comments** |  |  |
| --- | --- | --- | --- |
|  | **General comments** | **Author’s response** | **Location of the amendment in the manuscript** |
| 1 | Please ensure that your manuscript meets PLOS ONE's style requirements, including those for file naming. | Now the manuscript file meets PLOS ONE’s style requirements. | Throughout the document. |
| 2 | Please ensure you have thoroughly discussed any potential limitations of this study within the Discussion section, including the impact of potentially confounding factors. | Corrected | In respective supporting documents. (Figures 1 – 3) |
| 3 | Please include captions for your Supporting Information files at the end of your manuscript, and update any in-text citations to match accordingly. | Corrected | In respective supporting documents. (Figures 1 – 3) |
| 4 | We note you have included a table to which you do not refer in the text of your manuscript. Please ensure that you refer to Table 1 in your text; if accepted, production will need this reference to link the reader to the Table. | Included. Thank you for pointing this to us. | Page 6, Results, Paragraph 1 |
| 5 | Please ensure that you refer to Figure 3 in your text as, if accepted, production will need this reference to link the reader to the figure. | Included. Thank you for pointing this to us. | Page 7, paragraph 3 |
|  | **Reviewer 1** |  |  |
|  | **Major issues** |  |  |
| 1 | Needs language revision – needs significant improvement on English | Addressed. | Throughout the document. |
| 2 | Not a clear research question/hypothesis. For instance, the stated goal is to describe multi morbidity however data is presented on association between multi-morbidity and mental health outcomes. It is not clear if mental health conditions is the outcome variable, if so, the hypothesis should be clearly stated. | Thank you for pointing out this comment. We have done the necessary changes. | Page 4, para 2 |
| 2 | The goal of the study is not clear. Is the goal to describe the characteristics of people diagnosed with NCD (i.e., age and sex [gender]?) or to describe the characteristics of people diagnosed with 2 or more NCDs? | Thank you for pointing out this mistake. It has now been corrected. | Page 4, paragraph 2 |
|  | *Sample size: Description of sample size estimation is not clear*   - If the study is descriptive in nature, what is the significance level for? Is this an analytical or descriptive study? | Apologies for the mistake made with the terminology. It has been corrected now. | Page 5, Para 4 |
|  | - Needs to provide rationale for not including all patients in the registry and limiting it to a sample size calculation. | All adult patients diagnosed with at least one NCD were eligible for inclusion. The sample size was calculated to be able to extract a finite number yet yielding a sufficient statistical power. | Page 5, Para 3 |
|  | - Were the people excluded different than those that remained in the study? | Exclusion was based either on an age lesser than 18 years, or being devoid of any NCD. Otherwise, the people shared similar characteristics. However, those between 18-20 years were found not to have any NCDs among the individuals selected. | Page 5, Para 2 |
|  | **Minor issues** |  |  |
|  | Data was collected only for individuals over 20 years of age but no rationale is provided for this selection. | Even if the data were originally collected considering the adult patients (above 18 years), the ones who were 18 and 19 years old were found not to have any NCDs. Hence, their records had to be excluded from the study since we are focusing on the patients with at least one NCD. Therefore, to be more concise, the ages were categorized by taking the data of the patients who were over 20 years old who satisfied the inclusion criteria of the study. | Page 5, Para 2 |
|  | *Abstract.* |  |  |
|  | - Conclusions not supported by data | Corrected. Kindly see if the conclusions fit the results now. | Page 2 |
|  | - Abstract needs to be rewritten | Done. | Page 2 |
|  | - Reorder the introduction | Done. | Page 2 |
|  | - Not clear why only regression for mental health. | Thanks for pointing out the lack of clarity. The effect of multimorbidity on mental health was one of the revelations. The other aspects are described in the full-text. The clarification is made on the abstract now. | Page 2 |
|  | *Figures and tables.* |  |  |
|  | - Figure 3 is not high resolution and not clear | Apologies about it and the figure has been redrawn. | It has been included as a separate eps file in respective supporting documents. |
|  | - No need for tables under figures 1 and 2 | Corrected and the edited figures have been attached. | Included as separate eps files in respective supporting documents. |
|  | *Introduction* |  |  |
|  | - Might need some reformatting of paragraphs | Reformatted | Throughout the document |
|  | *Methods* |  |  |
|  | - Selection of clinic/hospital needs to be better justified (is it representative of the population, why? If not, how to avoid bias) | The Medical School of the University has only got Primary Care (University Family Practice Centre) and the Tertiary Care (University Medical Unit at the Teaching Hospital) that have collaborated here. Patient care coordination is shared between these two departments and secondary level hospital involvement is not routinely occurring. The clarifications are made in the manuscript. | Page 4-5 |
|  | - Again, provide rationale for selecting only individuals over 20 years. | The data were originally collected considering the patients over 18 years but the ones who were 18 and 19 years old were found to not have any NCDs in our sample. Hence, their records had to be excluded from the study pertaining to our exclusion criteria. Therefore, to be more concise, the ages were categorized by taking the data of the patients who were over 20 years old who satisfied the inclusion criteria of the study. | Page 5, Para 2 |
|  | - Needs to add rationale behind selecting diagnosis for inclusion. | Apologies for the mistake. The NCDs listed were the ones observed. However, all NCD was considered for inclusion and hence, the list is now removed from the text. | Page 5, Para 2 |
|  | - Formatting of age groups is different | Reformatted according to the given guidelines | Page 5, Para 3 |
|  | - Provide rationale for age groups | It is established in literature that the prevalence of multimorbidity substantially increased with age. Hence, our main intention was to categorize them into the minimum number of groups which would best interpret the age-specific analyses. Thus, the groupings were created with four age categories such that the ages included in the study were covered. | Page 5, Para 3 |
|  | *Methods.* |  |  |
|  | - Sample Size | The sample sizes for each of the four age groups were calculated using  n= Z^2^ p q/e^2^  with a prevalence (p) of 50% and 5% margin of error (e) with Z-score as 1.96 at 95% confidence level. | Page 5, Para 4 |
|  | - Is the data extraction form provided? | The supporting document is now included. (S1 Annexure Data Extraction Form) | Page 5, Para 4 |
|  | *Data analysis* |  |  |
|  | - Why restricting to age 18 and older when the eligibility states age 20 and older? | Apologies for the mistake. We would consider those aged above 20. This has now been corrected. | Page 6, Para 1 |
|  | - Suggest to use either SPSS or R. Indicate version, year, etc. full software citation. | - IBM SPSS Statistics Version 20 IBM Corp. (2017). *IBM SPSS Statistics for Windows*. Armonk, NY: IBM Corp. Retrieved from https://hadoop.apache.org - R version 3.3.3 (2017-03-06) RStudio Version 1.3.959 © 2009-2020 RStudio, PBC "Middlemist Red" (3a09be39, 2020-05-18) for Windows Mozilla/5.0 (Windows NT 10.0; Win64; x64) AppleWebKit/ 537.36 (KHTML, like Gecko) QtWebEngine/5.12.6 Chrome/69.0.3497.128 Safari/537.36 | Page 6, Para 3 |
|  | *Results* |  |  |
|  | - Not clear what is meant by “by the time they reach half the decade” – it would be best to describe the age group. | Appreciate pointing this out to us. It has been corrected. | Page 7, Para 1 |
|  | - The use of “increase” in line 148 suggests a baseline. I suggest to consider use another term that more accurately describes the comparision being made. | Appreciate pointing this out to us. This has been rectified. | Page 7, para 2 |
|  | - Use n= XXX as opposed to XX/1600 | Corrected as appropriate throughout the document. | Pages 2-9 |
|  | - How does the proportion of multimorbidity relate to original size of age groups (an adjusted rate might be useful)? | Thank you for pointing this out. We were unable to conduct an age standardised rate, as the data of the standard population in Sri Lanka for Colombo District is old (2011) and further other data like multimorbidity in a Population is yet not published. | Page 7, Para 1 |
|  | *Discussion* |  |  |
|  | - Could be more focused. | Thank you for pointing this out. We have tried our best to be focused and have done the changes throughout the manuscript to be focused. | Changes made throughout the manuscript. |
|  | - Needs to better discuss the strengths and limitations of the study. | A section is now added on strengths and limitations. | Page 12,13 |
|  | **Reviewer 2** |  |  |
|  | - Methods section: Please add subsections and reorganize as necessary to improve clarity and readability | Sub-sections are added now | Page 4-6 |
|  | - Methods (line 109) please justify why these age categories were used, if possible referencing previous studies for compatibility | Our main intention was to categorize them into the minimum number of groups which would best interpret the age-specific analyses. The optimal number of age groups was decided as four in our study since the majority of the patients were found to be elderly (over 50 years). | Page 5, Para 2 |
|  | - Methods (line 128) states the analysis was restricted "to those aged 18 years and older", however line 101 states "patients over 20 years ...". Please correct or clarify. | Apologies for the mistake. We would consider those aged above 20. This has now been corrected. | Page 6, Para 1 |
|  | - Table 2 Please clarify in the footnote which covariates were uncluded in the adjusted OR model. Would it be possible to present unadjusted and adjusted. Could additional factors be included in the adjusted model? | The details have been included in the footnote. Unadjusted/crude odds ratio are obtained when the effect of only one predictor variable is taken into consideration. However, in our study, the requirement was to include the bio-physical variables along with our variable of interest which is multimorbidity in order to make conclusions regarding their influence on the mental disorders. Hence, Adjusted Odds Ratios have been used which are computed by adjusting based on the confounding variables in the analysis.  All the variables in the study have been included in the model except for the types of non-communicable diseases since we are focusing only on the mental health disorders here using the logistic regression model. | Page 9 |
|  | - It is clear ethical approval was obtained from the relevant institute. Patient data was anonymous. Did patients give permission to the institute to use their data? | Thank you for expressing your concern. Since both the institutions belong to the University (Teaching Hospital), the patients are notified at the point of registration about the possibility that anonymized information from their clinical records being utilized for research purposes unless they specifically object towards same. |  |
|  | - Please carefully check English language usage throughout the text. | Addressed. | Throughout the document. |
