# Supplementary material for: The rising complexity and burden of multimorbidity in a middle-income country
Source: PLoS One. 2020 Dec 11;15(12):e0243614. doi: 10.1371/journal.pone.0243614 (PMC7732070; doi:10.1371/journal.pone.0243614)
Supplement: Attachment — Submitted filename: 01.11.20 Response to reviewers 2.docx [file pone.0243614.s006.docx]

**Response to the reviewers**

|  | **Comments** |  |  |
| --- | --- | --- | --- |
|  | **General comments** | **Author’s response** | **Location of the amendment in the manuscript** |
| 1 | Reviewer #2: Thank you for addressing almost all comments. Please additionally add subsection headers within the methods section. | Noted with thanks. |  |
| 2 | Reviewer #3: Examples of literature on MM in Intro and Discussion are largely from high income countries - when there are a reasonable number of publications from lower income countries that might be more appropriate. | Thanks a lot for the valuable suggestion. Added a few more references from LMICs (References from Asia/ LMIC countries. | Page 3, 9, 11  Lines 67-70, 188, 244, 240, 256- 257  References  8, 9, 10  21, 26, 28, 29, 31, 32, 33, 34 |
| 3 | Table 1 is useful, but could be more useful to have info by age and sex, plus a similar table of most common multimorbidities. Would want table of patient sociodemographic characteristics. The authors could please specify which variables were included in the adjusted regression analyses? | Thank you for the suggestions.  The socio-demographic details are illustrated as Table 1.  Table 1 in the previous draft has been renamed to Table 2 which consists of the additional columns for the variables age and sex.  Another table (Table 3) for the most common multimorbidities (affected number >100) as suggested has been included in the manuscript along with Figure 3, a pictorial representation of the multimorbidities with 2 disorders.  The variables included in the adjusted logistic regression model have been included in the footnote. | Table 1 - Page 07  Lines 153-154  Table 2 - Pages 07&08  Lines 155-156  Table 3 - Page 09  Lines 176-177  Table 4 - The regression output is now in Table 4 with more details and the covariates are stated in the footnote - Page 10  Lines 185-186 |
| 4 | More details about the regression analyses are needed - describing the stepped process by which you did both sets of regression analyses - and especially the analysis including mental health variables. | Thank you and a separate document with the detailed process of the binary logistic model fitted with the mental health variable as the outcome has been attached.  The output in Table 4 was also reformed with more details. | A separate document |
| 5 | And the conclusions about mental health need to be tempered - this is a cross-sectional study "...as the number of disorders increases, the risk of developing any mental disorders increases..." | Thanks for the suggestion. Evidence from the Asian region that well-aligns with our findings is now incorporated. | Please see the paragraph between lines 244 -255 |
| 6 | The term "elderly" is considered pejorative, please instead use older persons, older adult, older population. | Point well noted. Changes made in all respective locations on the manuscript. | (Lines 146, 199) |
| 7 | One of the biggest findings here is the high prevalence in 'young" age groups. This could be part of another analysis - (narrower age groups (20-29, 30-39, etc.) and look at age of diagnosis - but for this publication, authors could emphasize this interesting finding about high MM prevalence in young age groups. | Appreciate your suggestion regarding the ancillary analysis.  In terms of this paper, the salient finding that younger population falls prey to multimorbidity has been highlighted under the following sections. (Please refer to the line numbers) | Please see  Lines between  157– 160 (Results)  Lines between  206 – 223. (Discussion) |
| 8 | Finally, the title is misleading - it describes the burden of multimorbidity in a sample of patients, not its complexities. | Appreciate your suggestion. The title is now amended as “The rising complexity and burden of multimorbidity in a middle-income country” | Page 1 (Lines 1-2) |
