# Supplementary material for: The rising complexity and burden of multimorbidity in a middle-income country
Source: PLoS One. 2020 Dec 11;15(12):e0243614. doi: 10.1371/journal.pone.0243614 (PMC7732070; doi:10.1371/journal.pone.0243614)
Supplement: Attachment — Submitted filename: Response to reviewers 13.11.20 - Version 3.docx [file pone.0243614.s007.docx]

**Response to the reviewers – round 3**

|  | **General comments** | **Author’s response** | **Location of the amendment in the manuscript** |
| --- | --- | --- | --- |
| 1 | I would just like to see some modifications to the tables so that they are easier to read. In all tables, it would be helpful to have the sample size shown within the column headers so that the denominator for each cell is clear. | Thank you for the suggestion and the sample size (n = 1600) has been included in the column headers which give the ‘Number of patients’ in Tables 1, 2 and 3.   - Table 1 - In the last column - Table 2 - Second column (from left) - Table 3 - Second column (from left) | Pages 7 - 10 |
| 2 | For Table 2, you should consider having a total column (where you can show the prevalence of each condition for the full sample) and then the prevalence stratified by each sex and age; you sort of have this but you need the denominator for each column and perhaps a bit more cleaning up. | Thank you.  A column with the total number has already been included in table 2 and 3 (second column) which shows the prevalence with respect to each condition. These values thus serve as the denominators for the age and gender stratified prevalence values. Since the total numbers differ by each disease, the inclusion of them in the column headers will not be possible.  Hence, in order to make the table clearer, the columns and rows have been separated with thicker borders hoping that it help to represent the total number affected and the age and gender prevalence values with respect to each disease in a better manner. | Page 7 - 9 |
| 3 | You may want to consider similar adjustments to Table 3. There is a fair bit of data in your study and anything you can do to help your readers understand it would be useful. |  | Page 10 |
